# Thermosonication Applied to Kiwi Peel: Impact on Nutritional and Microbiological Indicators

**DOI:** 10.3390/foods12030622

**Published:** 2023-02-01

**Authors:** Magali Boghossian, María Emilia Brassesco, Fátima A. Miller, Cristina L. M. Silva, Teresa R. S. Brandão

**Affiliations:** CBQF—Centro de Biotecnologia e Química Fina—Laboratório Associado, Escola Superior de Biotecnologia, Universidade Católica Portuguesa, Rua Diogo Botelho 1327, 4169-005 Porto, Portugal

**Keywords:** fruit waste, *Listeria innocua*, mild thermal processes, proteins, fibers, minerals, chlorophylls, phenolics

## Abstract

The peels of many fruits are rich sources of nutrients, although they are not commonly consumed. If they are properly decontaminated, they can be used as healthy food ingredients reducing food waste. The objective was to apply thermosonication processes to kiwi peel and evaluate the impact on *Listeria innocua* survival (a non-pathogenic surrogate of *L. monocytogenes*) and key nutrients and quality indicators: proteins, fibers, minerals (Ca, K, Mg, Na, and P), chlorophylls, and phenolic contents. Kiwi peels were artificially inoculated with *L. innocua* and thermal and thermosonication treatments were performed at 55 °C and 60 °C for 30 and 15 min maximum, respectively. Bacteria were enumerated through treatment time, and quality indicators were assessed before and at the end of treatments. A Weibull model with a decimal reduction time (D-value) was successfully used in *L. innocua* survival data fits. Results showed that coupling temperature to ultrasound had a synergistic effect on bacteria inactivation with significant decreases in D-values. Thermosonication at 60 °C was the most effective in terms of protein, fiber, chlorophylls, and phenolics retention. Minerals were not significantly affected by all treatments. Applying thermosonication to kiwi peel was more effective for decontamination than thermal treatments at the same temperature while allowing the retention of healthy compounds.

## 1. Introduction

**Figure 1:**
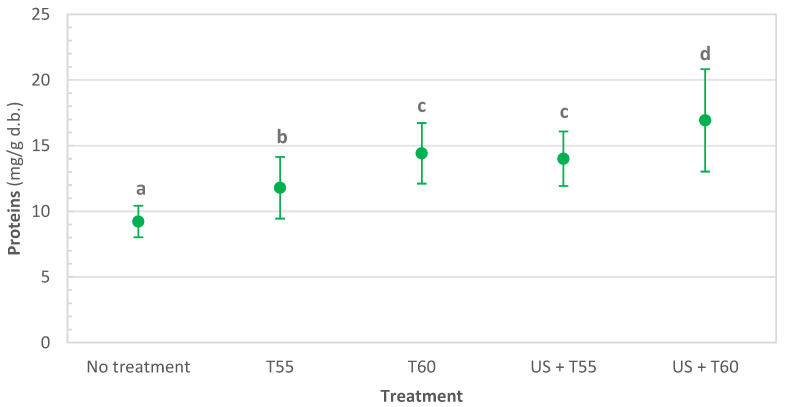
Protein content in untreated, thermally treated and thermosonicated kiwi peel. Values with different letters differ significantly (*p <* 0.01).

Food waste has reached substantial figures [1] being the environment highly affected. In the particular case of fruits, the processing industries discard huge amounts of materials, e.g., peels/rinds, seeds, and pomace, that are rich in nutrients and bioactive compounds [2,3,4]. If these products are conveniently transformed into edible forms, there will be potential waste reuse coupled with food scraps reduction. In this context, processing fruit residues to obtain microbiologically safe products is very important [5]. Less severe heat treatments at temperatures ranging from 30 to 60 °C have emerged in food processing, avoiding the degradation of quality-related nutrients, such as natural bioactive compounds, that are heat-sensitive. Some methodologies have been applied to improve the quality and safety of food products; high hydrostatic pressure, pulsed electric fields, ultrasound, and cold plasma are examples [6]. Among these processes, ultrasound plays an important role, particularly when coupled with mild thermal treatments (i.e., thermosonication). Due to a cavitation phenomenon, gas bubbles are generated as a result of rapid temperature and pressure changes. These bubbles expand, then implode and collapse, resulting in energy release and cellular damage to microorganisms. However, undesirable physicochemical modifications in the food may occur if the process is not adequately controlled [7,8,9,10]. This originates microorganisms and enzymes inactivation, being thermosonication more effective than ultrasound or heat treatments applied individually [11]. Furthermore, since the temperatures used in thermosonication processes are lower than the ones often applied in pasteurization, the impact on quality losses is reduced [12].

Thermosonication has been effective in achieving microbial and enzymatic inactivation while preserving the quality of many fruit juices, such as apple juice [13,14], grapefruit juice [15], and orange juice [16]. In the case of fruits by-products and residues, ultrasound, in combination with other processes, has been used as a pre-treatment before drying of passion fruit peel [17], and grape skin [18] or as an aid to extract compounds such as pectin from tomato waste and orange peel [19,20], phytocompounds from dragon fruit peel [21], flavonoids from kiwi peel [22], and anthocyanins from black carrot pomace [23]. However, as far as knowledge is concerned, studies of the impact of ultrasounds on microbial contaminants and quality features of fruit waste have not been carried out yet.

In this work, the target study was thermosonication applied to the kiwifruit peel. The peel contains high amounts of health-beneficial compounds such as vitamins C and E, chlorophylls, polyphenols, and flavonoids, all of which contribute to its antioxidant activity [24,25,26]. It is also rich in minerals such as potassium, magnesium, calcium, and phosphorus [27,28]. In the food industry, kiwifruit is processed mainly to produce desserts and beverages, and the peel is usually discarded. Kiwi peel is hairy, a characteristic that makes it unappealing to consume despite being perfectly edible [27]. Few studies have focused on kiwi peel, yet it was found to have a high nutritional value and a diversity of bioactive compounds.

Kiwifruit waste products are mainly used as fertilizers; however, they have the potential to create functional food products, especially peel [28]. Due to the characteristics of the kiwi’s external surface, microbial contaminants such as *Salmonella* spp., *Escherichia coli*, and *L. monocytogenes* can attach and survive, being potential sources of foodborne diseases [29]. For that, more research is needed on the composition of kiwi peel and on developing adequate processes for sanitizing, preservation and transformation into convenient edible forms. This opens opportunities to investigate the influence of ultrasound-based processes on kiwi peel contaminants, aiming to minimize the process’s impact on key-quality attributes and nutrients.

The objective of the work was to study the anti-listeria effect of thermosonication on kiwi peel by considering *Listeria* as a target contaminant. In addition, quality indicators such as bioactive compounds and some nutrients were evaluated. The overall goal was to assess the impact of thermosonication on kiwi peel decontamination and quality retention, which is innovative. Kiwi peel is not usually consumed, but a value-added ingredient may be created if conveniently processed and transformed.

## 2. Materials and Methods

### 2.1. Kiwi Peel Samples

Kiwis (*Actinidia deliciosa cv. Hayward*) were purchased at local markets in Porto, Portugal. They were acquired at the commercial maturity stage with no visual defects and stored in refrigerated conditions (5 ± 2 °C). Before analysis and treatments, the kiwi surfaces were rubbed gently with gauze to remove excess fuzz and smooth them. Peel was manually removed using a stainless-steel peeler and cut into small pieces of around 0.5 × 0.5 cm.

The moisture content of kiwi peel was evaluated according to the methodology recommended by the Association of Analytical Chemistry (Method 984.25; AOAC 2002 [30]).

### 2.2. Thermal and Thermosonication Treatments

Kiwi peels were thermosonicated using a stainless-steel ultrasonic bath containing a built-in heating device (Bandelin Sonorex, RK 102H, Berlin, Germany) with a capacity of 3 L, an ultrasound frequency of 35 kHz, heating power of 140 W, and maximum output power of 480 W. Thermosonication treatments were performed at 55 °C (US + T55) for 30 min and 60 °C (US + T60) for 15 min. At 55 °C, samples were removed after 5, 15, and 30 min of treatment; at 60 °C the sampling times were 5, 10, and 15 min. These temperatures were selected within the range usually assumed for mild heat treatments; the maximum sampling times were the ones that allowed obtaining at least 5-log cycles reduction for *L. innocua*.

Thermal treatments without ultrasound were performed at the same temperatures, 55 °C (T55) and 60 °C (T60), and sampling times. The ratio between sample mass and water volume was approximately 33 g L^−1^. The temperature of the bath was controlled using a digital thermometer.

*L. innocua* on kiwifruit peels was enumerated before and after each treatment at the three sampling times. Nutritional indicators (proteins, dietary fibers, and minerals—Ca, K, Mg, Na, P) and bioactive compounds (chlorophylls and total phenolics) were determined in fresh-cut kiwi peels and at the end of each treatment. Each treatment was repeated three times.

### 2.3. Nutritional Compounds

#### 2.3.1. Proteins

The protein percentage in kiwi peel was determined using the Kjeldahl method of nitrogen determination, assuming a factor of 6.25 for protein conversion [31].

Results were expressed in mg/g on a dry basis (d.b.) by multiplying the percentage obtained with the initial mass of the dried sample. The determination of proteins was done in triplicate.

#### 2.3.2. Dietary Fibers

Analyses of the total, soluble, and insoluble dietary fibers were performed using an enzymatic-gravimetric methodology. A dietary fiber kit (Megazyme, Wicklow, Ireland) based on AOAC method 991.43 and AACC method 32-7.01 [32] was used. Total dietary fiber content was the sum of soluble and insoluble dietary fibers obtained.

Results were expressed in mg/g on a dry basis by multiplying the results with the initial mass of the dried sample. The determination of dietary fibers was done in triplicate.

#### 2.3.3. Minerals

Minerals determination in kiwi peel followed the procedure described by Chatelain et al. [33], which is based on sequential digestion steps. Calcium (Ca), potassium (K), magnesium (Mg), sodium (Na), and phosphorus (P) concentrations were assessed using the inductively coupled plasma optical emission spectrometer (PerkinElmer^®^ 7000 DV). Results were expressed in mg/g on a dry basis. The analyses were performed in triplicate.

### 2.4. Bioactive Compounds

#### 2.4.1. Total Chlorophylls

Chlorophylls in kiwi peel were extracted following the procedure described by Fundo et al. [3]. Total chlorophylls were the sum of chlorophylls a and b, determined by spectroscopy [34]. Results were expressed as µg/g on a dry basis. Measurements were made in triplicate.

#### 2.4.2. Total Phenolics

The total phenolic content in kiwi peel was determined as described by Fundo et al. [3], using the Folin-Ciocalteu reagent.

Data were expressed in mg of gallic acid equivalents (GAE) per g of sample on a dry basis (mg/g). The determination of total phenolic content was done in triplicate.

### 2.5. L. innocua Enumeration

*L. innocua* 2030c was obtained from the private collection of the Public Health Laboratory Service (Colindale, UK). Sub-cultures were prepared as described by Miller et al. [35] and suspensions of approximately 10^8^ CFU/mL of the bacterium were used for inoculation purposes. To artificially inoculate kiwi peels with *L. innocua*, each sample of 5 g was immersed for 15 min in 20 mL of the bacteria suspensions previously prepared. Samples were left to air for 15 min until surface drying.

After inoculation, and before and after each treatment applied, 45 mL of buffered peptone water (BPW, Lab M, Lancashire, UK) was added to 5 g of kiwi peel to stomacher bags and mixed for 2 min. Samples were serially diluted with BPW and plated in duplicate onto Palcam agar containing a PALCAM Selective Supplement (Biokar diagnostics, Beauvais, France). This supplement favors the growth of *Listeria* while inhibiting the growth of other contaminating microorganisms. Plates were incubated at 37 °C, and bacteria were counted daily until the number of colony-forming units (CFU) was no longer increased.

### 2.6. Modeling of L. innocua Survival

A Weibull model was used to fit *L. innocua* survival data obtained after thermosonication and thermal treatments [36]:

(1) $$\log\left(\frac{N}{N_{0}}\right)=-{\left(\frac{t}{D}\right)}^{n}$$

N represents the microbial load after treatment (CFU/mL), N_0_ represents the sample’s initial microbial load (CFU/mL), t is the treatment time (min), D is the first decimal reduction time, i.e., the time required to achieve the first 1-log reduction (min), and n is a shape parameter (dimensionless). A shape parameter n < 1 indicates upwards concavity, n > 1 indicates downward concavity, and n = 1 indicates linearity.

The model expressed in eq. 1 was fitted to experimental data by non-linear regression analysis using IBM SPSS Statistics for Windows, Version 27.0 (SPSS Inc., Armonk, NY, USA).

Residual analyses assessed the model adequacy. Residuals (i.e., differences between experimental values and values estimated by the model) were tested for their randomness and normality (with mean equal to zero and constant variance). Randomness was verified by visual inspection of the distribution of residuals versus values predicted by the model and normality by the Shapiro-Wilk test.

The coefficient of determination R^2^ was calculated as a measurement of the proportion of the variance in *Listeria* survival that is predictable from the model assumed. The goodness of fit occurs when values are close to 1.

### 2.7. Statistica Analyses

One-way ANOVA assessed the effect of treatments on the quality indicators with a post-hoc Duncan test for means comparisons. The requirements of normality and homoscedasticity of data within groups were tested using Shapiro-Wilk and Levene’s tests, respectively. A significant level of 1% was assumed in all tests performed. Results were expressed as mean ± half of the confidence interval at 95%, except for the characterization of fresh kiwifruit peel. In this case, results were expressed as mean ± standard deviation.

IBM SPSS Statistics for Windows, version 27.0 (SPSS Inc., Armonk, NY, USA) was used in all statistical analyses.

## 3. Results and Discussion

Results of the characterization of kiwi peel in terms of moisture content, proteins, dietary fibers, minerals, chlorophylls, and total phenolic content are included in Table 1. The rationale for choosing these specific compounds and nutrients is their abundance in kiwi peel, being multiple health benefits associated with their ingestion. Moisture in the peel amounted to 76.74 ± 0.76%, which is a favorable condition for the growth and survival of microorganisms. Protein content was 4.54 ± 0.23%. Results obtained by Salama et al. [25] showed a higher moisture content (85.27%) and a higher protein content (12.62%). Soquetta et al. [24] reported that protein content in dried kiwi peel ranged from 3.84 to 8.31%. The disparity in results may be explained by different fruit maturity levels, varieties or geographic conditions [37]. Fruits and their non-edible parts are a good source of dietary fibers, which are essential for the functioning of the human body. In the case of kiwi peel, the total fiber content was found to be 30.45 ± 0.40%, most of which is insoluble (29.68 ± 0.17%). The minerals analyzed in kiwi peel were P, Mg, Ca, Na, and K, being potassium the most abundant one amounting to 93.94 ± 12.28 mg/g d.b. These results differ from those obtained by Salama et al. [25] in which Mg was the most relevant mineral. As previously remarked, varieties and different maturity stages may explain this issue. Kiwifruit peel and seeds have a similar composition [28], being important sources of essential and healthy nutrients that deserve valorization.

### 3.1. Treatment Effects on Nutritional and Bioactive Compounds

The temperatures of the treatments were monitored in all experiments. The values were 55 ± 1 °C and 60 ± 1 °C.

Results obtained for protein content in fresh (untreated), thermally treated, and thermosonicated kiwi peel are depicted in Figure 1. Protein content increased by 28%, 56%, 52%, and 84% after T55, T60, US + T55, and US + T60, respectively. However, US + T55 and T60 were not statistically different. The combination of the US with the temperature of 60 °C was the most effective treatment since it resulted in the greatest increase in protein content, which amounted to 16.93 ± 3.90 mg/g d.b., corresponding to 8.22%. Karki et al. [38] used high-intensity ultrasound in soy flakes, and results showed a proportional increase in protein release into extract; protein yield increased by 46%. This could be attributed to ultrasound’s cavitation effect, which breaks down the cells and hence helps in protein extraction [39].

**Figure 2:**
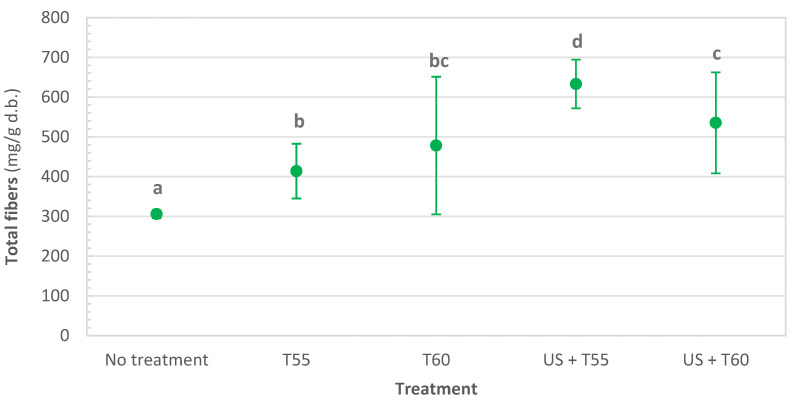
Total dietary fibers in untreated, thermally treated and thermosonicated kiwi peel. Values with different letters differ significantly (*p <* 0.01).

All treatments also significantly and positively impacted total fiber content (Figure 2). Thermal treatments (T55 and T60) were not statistically different but allowed an average increase of 46% in total fiber content compared to fresh samples. After thermosonication, the content also increased significantly, up to 75% after US + T60 and doubling after US + T55. The difference between thermal treatment at 60 °C (T60) and thermosonication at the same temperature (US + T60) was not significant. Similar to proteins, ultrasound, due to the cavitation effect, leads to an increase in the extraction yield of dietary fibers [40]. This may also explain the increase in dietary fiber content in thermosonicated kiwi peel. Published results showed that treating grapefruit peel at 70 °C for 25 min at 20 kHz allowed an 88% increase in the dietary fiber extraction yield and a reduction of 17% in the treatment time [41].

Minerals were determined only in fresh kiwi peel and after thermosonication treatments (Table 2). Mineral composition of kiwi peel was not significantly affected by thermosonication. This means that thermosonication at both temperatures and treatment times allowed mineral compounds’ retention. In terms of the impact of thermosonication on minerals, no studies were found. However, sonicating grapefruit juice at 28 kHz at room temperature (20 °C) resulted in a significant increase in Na, K, Ca, and Zn and a significant decrease in Mg [15].

**Figure 3:**
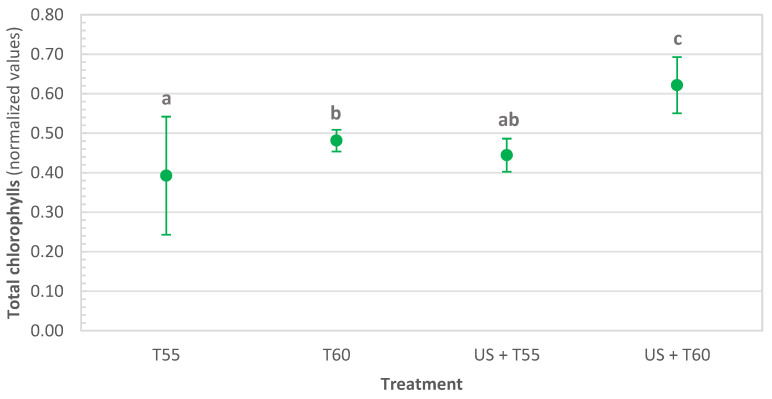
Effect of thermal and thermosonication treatments on total chlorophylls in kiwi peel (normalized values in relation to untreated samples). Values with different letters differ significantly (*p <* 0.01).

Treatment effects on total chlorophylls are shown in Figure 3. There was great variability in total chlorophylls amount detected in the fresh peel, ranging between 98.43 µg/g d.b. and 317.89 µg/g d.b. Hence, results were normalized in relation to the values obtained for fresh/untreated samples to be able to check the impact of treatments. Total chlorophyll in fresh samples decreased significantly after all treatments. However, US + T60 allowed the highest retention (62.1%) followed by T60 (48.1%), US + T55 (44.4%), and T55 (39.2%). Cruz et al. [42] reported no chlorophyll degradation when applying thermosonication to watercress. However, treatment time, temperature applied, and ultrasound intensity differed from the ones used in this work. Furthermore, thermal treatments significantly decreased chlorophyll content, such as in broccoli [43] and wheatgrass juice [44].

**Figure 4:**
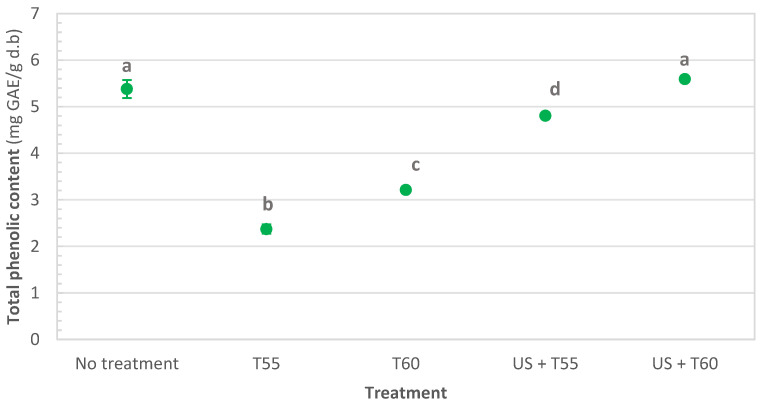
Total phenolic content in untreated, thermally treated, and thermosonicated kiwi peel). Values with different letters differ significantly (*p <* 0.01).

As for total phenolics (Figure 4), thermosonication at 60 °C allowed their total retention, while significant decreases of 11, 40, and 56% were observed after US + T55, T60, and T55, respectively. Analysis of phenolic content in whole tomatoes showed that they increased by 14% after ultrasonication and decreased by 14% and 12%, respectively, after heat treatment and thermosonication at 40 °C for 30 min [45]. Rawson et al. [46] applied thermosonication to watermelon juice and found that total phenolics significantly decreased after 10 min of treatment at 45 °C. However, Jabbar et al. [47] confirmed that although some losses of phenols occurred after the thermosonication of carrot juice, higher retention was achieved when compared to thermal treatments.

In general, ultrasound is linked to cellular tissue damage [48]. The release of ionic compounds (i.e., smaller molecules such as minerals) is associated with membrane damage. In contrast, higher molecular weight compounds (i.e., polyphenols and other biocompounds) also require cell wall fractures [49]. The disintegration degree and efficiency of bioactive compounds extraction in apple, banana, and persimmon skin cells induced by ultrasound was reported by Wang et al. [49].

### 3.2. L. innocua Survival

**Figure 5:**
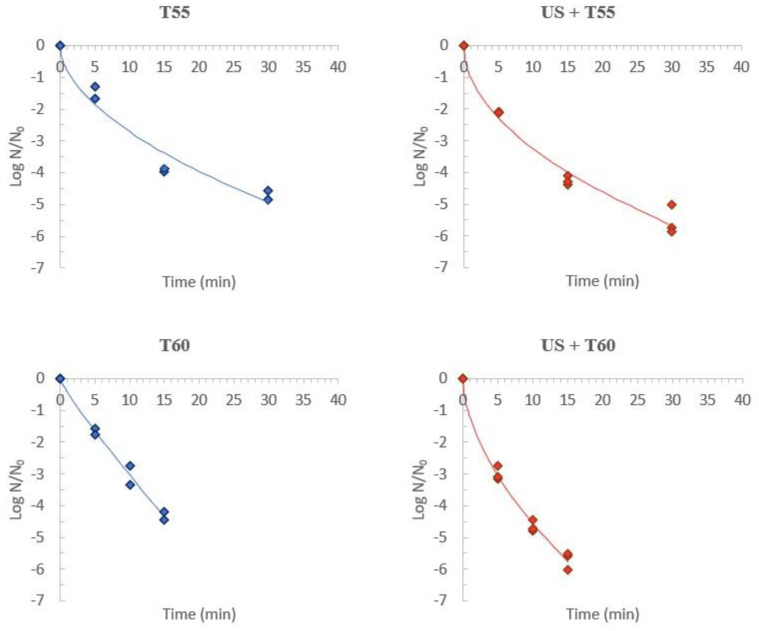
*L. innocua* survival in kiwi peel treated with thermal and thermosonication processes. Points correspond to the experimental data and lines represent fits of the Weibull model.

*L. innocua* survival was evaluated in kiwi peel before and after the treatments applied. This microorganism was chosen as a non-pathogenic surrogate of *L. monocytogenes*, which is often used as an indicator of thermal processes’ efficiency [35]. Results of *L. innocua* inactivation in kiwi peel are included in Figure 5. The mean value of initial surface contamination was 7.0 ± 0.10 log-cycles, and the results were normalized in relation to initial counts to avoid the influence of initial contamination. Treating kiwi peel at 55 °C for 30 min (T55) allowed a 4.7 ± 0.22 log reduction in *L. innocua*, while treatments at 60 °C (T60) for 15 min allowed a 4.3 ± 0.20 log reduction. To attain approximately 5 log-cycles of inactivation, the processing time at 60 °C was half of the one observed at 55 °C. When sonication was coupled to temperature, 5.5 ± 0.47 log reductions were achieved at 55 °C (US + T55) after 30 min, and 5.7 ± 0.28 log reductions were achieved at 60 °C (US + T60) after 15 min. Thermosonication at both temperatures and at the maximum treatment time allowed approximately 1 log-cycle more of *L. innocua* inactivation compared to the thermal processes at the same conditions.

The Weibull model was successfully fitted to *L. innocua* survival data for all treatments applied (Figure 5). Residuals were random and normally distributed, with mean equal to zero and constant variance (data not shown). Estimated D-values were 1.60 ± 1.71 min (T55) and 2.82 ± 0.90 min (T60). When thermosonication was used, those values decreased, respectively, to 0.97 ± 0.54 min and 0.73 ± 0.29 min. This shows that temperature coupled to ultrasound was more effective in *L. innocua* inactivation than without ultrasound. To attain the first 1-log cycle reduction, less treatment time was required when thermosonication was applied. In the case of fruit juices, the Food and Drug Administration [50] set up 5-log reductions of target microorganisms to attain a stable processed product. Besides fruit peels are not included in this criterium, this value was used to predict adequate processing times and compare the treatments under study.

The Weibull model with the estimated parameters (Table 3) was used to calculate processing times to achieve a 5-log reduction. The times were 30.5, 23.4, 17.7, and 11.8 min for T55, US + T55, T60, and US + T60, respectively. Based on these results, the experiments performed to study the impact of thermal and thermosonication processes on quality indicators were set at 30 min for treatments at 55 °C (with and without ultrasound) and 15 min for treatments at 60 °C (with and without ultrasound). These processing times guarantee a stable product from a microbiological perspective, and the impact on quality was posteriorly assessed. It can be concluded that US + T60 is the preferable treatment to inactivate *L. innocua* in kiwi peel; the smallest first D-value and the shortest time required to attain a 5-log reduction corroborate these findings. Throughout 15 min of treatment, the behavior of listeria survival at T60 was similar to that observed at US + T55. This means that a mild process in terms of temperature can be used to process the kiwifruit peel.

Combining temperature with ultrasound was proven to be even more effective in reaching microbial reductions beyond 5-log cycles, especially in liquid foods such as fruit and vegetable juices [51].

It is important to note that the thermosonication effectiveness in inactivating microorganisms depends on many factors, such as food matrix and its physicochemical characteristics, target microorganisms, temperature, treatment time, ultrasound power, and frequency. For example, treating mango juice at 60 °C for 7 min at 25 kHz resulted in a 5-log reduction in *Escherichia coli* O157H:7 [52]; treating orange juice at 55 °C for 30 min at 30 kHz resulted in a 5.5-log reduction in *Staphylococcus aureus* [53]. Alexandre et al. [54] studied the impact of thermosonication at 50, 55, 60, and 65 °C on the following microbial loads: *L. innocua* inoculated (in red bell peppers), total endogenous mesophiles (in strawberries), and total coliforms (in watercress). Thermosonication and heat treatment at 50, 55, and 60 °C allowed similar microbial inactivation in all the products studied. For total coliforms/watercress at 65 °C, thermosonication had a higher impact on coliforms reduction.

## 4. Conclusions

Combining ultrasound with temperatures of 55 °C and 60 °C showed a synergistic effect in *L. innocua* inactivation. At 55 °C, treating the peel with ultrasound for 30 min allowed an additional 0.8 log-cycle reduction compared to a thermal treatment at the same temperature; and at 60 °C for 15 min, an additional 1.4 log-cycle reduction was achieved. The Weibull model was successfully used to fit *L. innocua* survival data, and the parameters estimated allowed for the calculation of the time required to attain a 5-log reduction for each treatment: 30.5, 23.4, 17.7, and 11.8 min were the times obtained, respectively for T55, US + T55, T60, and US + T60.

Thermosonication treatments were more effective than thermal treatments in retaining nutrients and quality parameters. After all treatments, protein content significantly increased by 28%, 56%, 52%, and 84%, respectively, for T55, T60, US + T55, and US + T60. Dietary fibers increased by 35% and 56% for T55 and T60. When sonication was applied, they increased to 75% after US + T60 and doubled after US + T55. Thermosonication also allowed the retention of the following minerals: potassium, calcium, phosphorus, magnesium, and sodium. Total chlorophyll decreased significantly after all treatments; however, US + T60 allowed the highest retention (62%). Total phenolics were retained after thermosonication at 60 °C, significantly reduced by 11, 40, and 56% after US + T55, T60, and T55, respectively.

Thermosonication is a successful alternative to conventional heat treatments since it ensures decontamination while allowing retention and even increase in essential healthy nutrients of kiwifruit peel. It is a promising technology for developing new and safe ingredients based on food waste.

## Figures and Tables

**Table 1:** Composition of the kiwi peel.

Item	Composition *
Moisture content (%)	76.74 ± 0.76
Proteins (%)	4.54 ± 0.23
Total dietary fibers	30.45 ± 0.40
Soluble fibers (%)	0.77 ± 0.23
Insoluble fibers (%)	29.68 ± 0.17
Minerals (mg/g d.b.)	
P	3.30 ± 0.36
Mg	3.15 ± 0.45
Ca	9.01 ± 1.20
Na	0.37 ± 0.10
K	93.84 ± 12.28
Total Chlorophylls (µg/g d.b.)	199.82 ± 88.26
Chlorophyll a (µg/g d.b.)	131.54 ± 52.75
Chlorophyll b (µg/g d.b.)	68.28 ± 37.09
Total Phenolics (mg/g d.b.)	5.38 ± 0.18

* Values are mean ± standard deviation.

**Table 2:** Mineral composition of untreated and thermosonicated kiwi peel.

Treatment	Mineral (mg/g d.b.) *				
	P	Mg	Ca	Na	K
No treatment	3.30 ± 0.91	3.15 ± 1.12	9.01 ± 2.98	0.37 ± 0.25	93.84 ± 30.76
US + T55	3.91 ± 0.56	3.16 ± 0.53	10.69 ± 3.33	0.33 ± 0.10	74.03 ± 13.21
US + T60	3.81 ± 0.62	2.88 ± 0.37	9.84 ± 0.95	0.42 ± 0.08	88.67 ± 4.84

* Values are mean ± half of the confidence intervals at 95%.

**Table 3:** Weibull model parameters and R^2^ obtained for the different treatments.

Treatment	D (min) *	N *	R^2^
T55	1.60 ± 1.71	0.54 ± 0.22	0.960
T60	2.82 ± 0.90	0.88 ± 0.19	0.989
US + T55	0.97 ± 0.54	0.51 ± 0.09	0.985
US + T60	0.73 ± 0.29	0.58 ± 0.08	0.993

* Values are mean ± half of the confidence intervals at 95%.

## Data Availability

Data is contained within the article.
